# Characterization of Proteolytic Activity of Artichoke (*Cynara scolymus* L.) Flower Extracts on Bovine Casein to Obtain Bioactive Peptides

**DOI:** 10.3390/ani10050914

**Published:** 2020-05-25

**Authors:** Estefanía Bueno-Gavilá, Adela Abellán, María Soledad Bermejo, Eva Salazar, José María Cayuela, David Prieto-Merino, Luis Tejada

**Affiliations:** 1 Department of Human Nutrition and Food Technology, Universidad Católica de Murcia UCAM, Campus de los Jerónimos, 30107 Guadalupe (Murcia), Spain; aabellan@ucam.edu (A.A.); marisolbercar@gmail.com (M.S.B.); esalazar@ucam.edu (E.S.); jmcayuela@ucam.edu (J.M.C.); ltejada@ucam.edu (L.T.); 2 Applied Statistical Methods in Medical Research Group, Universidad Católica de Murcia UCAM, Campus de los Jerónimos, 30107 Guadalupe (Murcia), Spain; dprieto@ucam.edu

**Keywords:** antioxidant, artichoke, bovine casein, cinarases, angiotensin converting enzyme (ACE), proteolysis

## Abstract

**Simple Summary:**

Recently, dairy proteins, in addition to their basic nutritional role in the diet, were recognized as a source of bioactive peptides. Such peptides are encoded within the primary structure of the protein and can be released by enzymatic hydrolysis. The growing interest in the development of functional foods for the benefit of consumer health led to a recent increase in research on the production of bioactive peptides from different matrices and production methods. The use of aspartic proteases from stigmas of mature artichoke (*Cynara scolymus* L.) flowers to obtain hydrolytic enzymes (cinarases) in the production of bioactive peptides would involve the utilization of an agricultural residue of a plant species of great socio-economic importance. In the present study, the characterization of the optimal hydrolysis conditions of artichoke flower extracts was carried out for the production of peptides from bovine casein. Furthermore, the angiotensin-converting enzyme-I inhibitory activity and the antioxidant capacity against 1,1-diphenyl-2-picrylhydrazyl (DPPH) and 2,2’-azino-bis (3-ethylbenzothiazoline-6-sulphonic acid (ABTS) free radicals in vitro were determined for the obtained hydrolysates. The results revealed that the water-soluble extract of artichoke flower could be suitable for the production of bioactive peptides from whole bovine casein.

**Abstract:**

The aim of this work is to establish the most suitable proteolysis conditions to obtain bovine casein hydrolysates containing peptides with antioxidant and antihypertensive capacity. To this end, the proteolytic activity of *Cynara scolymus* L. flower extracts was characterized on whole bovine casein, evaluating the effect of several factors (pH, temperature, substrate concentration, enzyme concentration, and hydrolysis time). The optimal conditions to carry out the hydrolysis with the *C. scolymus* L. extract were as follows: pH 6.2, 50 °C, and 0.023 mg·mL^−1^ of extract-protein concentration. A Michaelis constant (K_m_) value of 5.66 mg·mL^−1^ and a maximum rate of reaction (V_max_) of 8.47 mUAbs∙min^−1^ were observed. The optimal hydrolysis time was 17 h. The casein hydrolysates obtained with these conditions contained peptides with antioxidant activity (1,1-diphenyl-2-picrylhydrazyl (DPPH) radical scavenging capacity: 30.89%; Trolox equivalent antioxidant capacity (TEAC) against 2,2’-azino-bis(3-ethylbenzothiazoline-6-sulphonic acid) free radical (ABTS^●+^): 4.43 mM Trolox equivalent·mg^−1^ peptide) and antihypertensive activity, showing 55.05% angiotensin-converting enzyme-I inhibition in vitro.

## 1. Introduction

Milk is considered the main source of bioactive peptides with specific nutritional, sensory, and functional properties [1]. During the last few years, the interest in the use of dairy hydrolysates with bioactive peptides increased for the maintenance of health and the prevention of chronic diseases. Numerous studies showed the bioactivity of peptides isolated from milk and dairy products [1,2,3,4,5].

In order to obtain bioactive peptides, different technologies were developed based mainly on enzymatic hydrolysis. To this end, enzymes of animal origin are used while the use of plant proteases is still limited [6]. The raw or lyophilized vegetable extract obtained from thistle flowers of different species, e.g., *Cynara cardunculus*, *Cynara humilis*, and *Carlina acanthifolia*, is successfully applied as a milk coagulant in the elaboration of sheep cheeses [7,8,9] and goat cheese [10,11]. In all these cases, a high proteolytic activity on caseins was demonstrated by the extract enzymes which could be attributed also to the impact of thistle coagulant on bacterial diversity and cheese dynamics [12].

Aspartic proteinases from the artichoke flower (*Cynara scolymus* L.) [13,14] were also isolated and characterized for the production of cheeses. It was observed in dairy products that these proteases, due to their specificity, produce a higher concentration of hydrophobic peptides [15,16] that present a greater inhibitory activity of the angiotensin converting enzyme-I (ACE-I) [17,18]. All these results indicate that it would be possible to use proteases from the artichoke flower to obtain hydrolysates rich in bioactive peptides, which would also entail the use of an abundant agricultural residue.

Optimal conditions were only studied for the use of proteases from *C. cardunculus* and *C. scolymus* in milk coagulation [13,19,20,21]. Silva and Malcata [22] defined a mathematical model to describe the proteolytic activity of cardosin A and B in ovine and caprine caseins. However, the proteolytic activity of *C. scolymus* flower extracts is not yet quantified, nor are the optimal conditions known which are necessary to obtain casein hydrolysates with a high content of peptides with potential bioactivity.

Therefore, the main objective of this work is to determine the proteolytic activity of *C. scolymus* flower extracts on bovine casein, evaluating the effect of pH, temperature, substrate concentration, enzyme concentration, and hydrolysis time. The aim is to establish the most adequate conditions to obtain hydrolysates containing peptides with antioxidant and antihypertensive capacity.

## 2. Materials and Methods 

### 2.1. Lyophilized *C. scolymus* L. Extract Preparation

Ripe artichoke flowers (*C. scolymus* L.) from the Region of Murcia (Spain) were used. The freeze-dried vegetable enzymatic extract was obtained following the procedure described by Tejada et al. [9]. From dried flowers of the *C. scolymus* L. species, styles and stigmas were isolated from the rest of the flower by scissoring the entire portion of flower protruding from the bracts. The plant material obtained was weighed and macerated at room temperature in distilled water 1:5 (*w*/*v*) for 24 h. After this time, the aqueous extract was sieved to remove impurities, and the filtrate was centrifuged at 3000 × *g* for 5 min. The obtained supernatant was filtered with Whatman paper nº1 under vacuum conditions. The resulting filtrates were frozen at −32 °C for 24 h and then lyophilized at a working pressure between 4 and 13 Pa. The freeze-dried powder obtained was hermetically packaged and kept frozen at −20 °C until use.

The mean protein content of the artichoke extract obtained, determined by the Bradford method [23], was 104 ± 10 (mean ± standard error) mg·mL^−1^. For every 100,000 mg of dried artichoke flower stigmas, an average of 1380 ± 280 (mean ± standard error) mg of protein was obtained in the freeze-dried extract.

### 2.2. Caseinolytic Activity Assay

The proteolytic activity of the enzymatic extract was determined following the method employed by Silva and Malcata [22]. The substrate used was bovine milk casein free of carbohydrates and fatty acids (Calbiochem, Darmstadt, Germany) at 1% (*w*/*v*) in 10 mM citrate buffer (pH 6.2) with 0.03% (*w*/*v*) sodium azide to avoid microbial growth, and it was incubated in a bath at 30 °C. Hydrolysis was started by adding 0.12 mL of extract inoculum (0.6 mg protein·mL^−1^) to 3 mL of casein solution. Afterward, 0.5-mL aliquots at different times (2, 5, 10, 20, 30, 40, 50, and 60 min) were taken in Eppendorf tubes. The enzymatic reaction was inactivated at 100 °C for 5 min, and the proteolytic activity was quantified by evaluation of the peptides soluble in aqueous 5% (*w*/*v*) trichloroacetic acid (TCA). For this purpose, 1 mL of 5% TCA (*w*/*v*) was added to each tube. The mix was incubated for 10 min at room temperature and centrifuged at 12,000 × *g* for 10 min, while the absorbance of the supernatant was measured at 280 nm in a quartz cuvette. All determinations were made in triplicate.

### 2.3. Temperature, pH, and Extract Concentration Optimization 

The effect of temperature, pH, protein concentration of enzymatic extract, and the hydrolysis time on proteolytic activity was analyzed using commercial bovine casein as a substrate. In all cases, the method described by Silva and Malcata [22] was followed, with modifications. For the study of the influence of these parameters on enzymatic kinetics, linear and polynomial (using square and cubic variables of explanatory variables) regression models were determined. The K_m_ value and the maximum velocity (V_max_) of the enzymatic reaction were estimated from the optimal conditions of hydrolysis.

In order to determine the temperature effect on the enzymatic activity, tubes with 3 mL of casein solution 1% in 10 mM citrate buffer (*w*/*v*) (pH 6.2) were incubated with 0.12 mL of the artichoke extract (0.6 mg protein·mL^−1^), at different temperatures: 22, 30, 40, 50, 60 and 70 °C.

To study the effect of pH, tubes with 3 mL of 1% (*w*/*v*) casein solution in 10 mM citrate buffer at different pH values (5.0, 5.5, 6.0) and phosphate buffer (pH 7.0 and 8.0) were incubated at 30 °C with 0.12 mL of the enzyme extract (0.6 mg protein·mL^−1^).

To analyze the enzyme linearity response, tubes with 3 mL of 1% casein dissolution in 10 mM citrate buffer (*w*/*v*) (pH 6.2) were incubated at 30 °C with 0.12 mL of different protein concentrations of artichoke extracts, in a range of 0.13–4.8 mg·mL^−1^.

For the study of each of these parameters, aliquots of 0.5 mL were taken at different times: 3, 6, 11, 21, 31, 41, 51, and 61 min. Proteolytic activity was quantified by measuring the 5% (*w*/*v*) TCA-soluble peptides adding 1 mL of 5% TCA (*w*/*v*) to each tube and incubating them for 10 min at room temperature. Then, the tubes were centrifuged at 12,000 × *g* for 10 min. The absorbance of the supernatant was read at 280 nm in a quartz cuvette. All determinations were made in triplicate.

### 2.4. V_max_ and K_m_ Estimation and 24-h Enzymatic Kinetic Evaluation

The determination of the kinetic parameters (V_max_ and K_m_) of the lyophilized artichoke extract was carried out using a concentration of extract protein in the inoculum of 0.6 mg·mL^−1^. As substrate, casein solutions in a range of 1.68–15 mg·mL^−1^ in 10 mM citrate buffer (*w*/*v*), pH 6.2, at 30 °C were used. Aliquots were collected at different reaction times: 2, 5, 10, 20, 30, 40, 50, and 60 min. 

To evaluate the proteolytic activity of the enzymatic extract over 24 h, tubes with 3 mL of a 1% casein solution in 10 mM citrate buffer (*w*/*v*), pH 6.2, preincubated at 30 °C were mixed with 0.12 mL of the enzymatic extract at a protein concentration of 0.6 mg·mL^−1^. Aliquots were collected, and the 5% (*w*/*v*) TCA-soluble peptides were measured at different reaction times over 24 hours. 

### 2.5. Casein Hydrolysate Preparation

To evaluate the production of bioactive peptides, casein hydrolysates were obtained at 17 h in triplicate. A 1% solution of bovine casein was prepared in distilled water (*w*/*v*) adjusting the pH to 6.2 with NaOH (0.1 N). The hydrolysis reaction was carried out at 50 °C with a protein concentration of the enzyme extract of 0.023 mg·mL^−1^ casein solution. The reaction was stopped at 17 h by increasing the temperature to 100 °C for 10 min, and the pH was adjusted to 4.6 with HCl. Subsequently, the hydrolysate was centrifuged at 4000 × *g* for 20 min, and the supernatant was collected and filtered through a 0.45-μm nylon filter. Finally, the pH of the permeate hydrolysate was adjusted to 7.0 with NaOH (1 N) and distributed in Falcon tubes for storage at −20 °C until use.

### 2.6. Angiotensin Converting Enzyme- I (ACE-I) Inhibitory Activity

The ACE-I inhibition of the hydrolysate was determined using the Cushman and Cheung spectrophotometric method [24] modified by Miguel et al. [25]. Briefly, each sample (0.040 mL) was incubated with 0.1 mL of 5 mM of hippuryl-histidyl-leucine (Sigma-Aldrich, St. Louis, MO, USA) dissolved in 0.1 M borate buffer (pH 8.3) containing 0.3 M NaCl. Then, 2 mU of ACE (EC 3.4.15.1, Sigma-Aldrich, St. Louis, MO, USA) was added, and the reaction mixture was incubated at 37 °C for 30 min. The reaction was stopped with 0.15 mL of 1 M HCl. The hippuric acid form was extracted by adding 1 mL of ethyl acetate, with the subsequent separation and evaporation of the organic phase. The residue obtained was resuspended in distilled water, and its absorbance (Abs) was measured at 228 nm. The ACE inhibitory activity was calculated using the following formula:

ACE inhibitory activity (%) = (Abs control − Abs sample)/(Abs control − Abs blank) × 100(1)

### 2.7. Antioxidant Activity

The antioxidant activity against 1,1-diphenyl-2-picrylhydrazyl (DPPH) was determined following the method described by Bersuder et al. [26]. Firstly, 0.5 mL of the hydrolysate was dissolved in 0.5 mL of ethanol and mixed with 0.125 mL of a solution of DPPH (Sigma-Aldrich, St. Louis, MO, USA) in ethanol (0.02% *w*/*v*). The mixture was incubated for 1 h in the dark and centrifuged at 10,000× *g* for 2 min. Absorbance was measured at 517 nm. The percentage of radical scavenging activity (RSA) was determined using the following formula:

DPPH RSA (%) = (Abs control − Abs sample)/Abs control × 100.(2)

The antioxidant activity against 2,2’-azino-bis(3-ethylbenzothiazoline-6-sulphonic acid) (ABTS) radical was determined using the method described by De Gobba et al. [27]. To produce ABTS radical (ABTS^•+^), a solution of ABTS diammonium salt (19.4 mM) (Sigma-Aldrich, St. Louis, MO, USA) with potassium persulfate (6.7 mM) in distilled water was prepared and left to react overnight. The ABTS^•+^ solution was then diluted 350 times with buffered phosphate (10 mM, pH 7.4) to achieve an absorbance of 0.6 to 0.7. The ABTS^•+^ working solution (0.2 mL) was mixed with 0.05 mL of the hydrolysate. Then, the absorbance was measured at 405 nm over 30 min. The final readings were used to calculate the ABTS^•+^ RSA (%) according to the following formula:

ABTS^•+^ RSA (%) = 100 − (100 × Abs sample/Abs control) (3)

The Trolox equivalent antioxidant capacity (TEAC) value of the hydrolysate (mM Trolox equivalent·mg^−1^ peptide) was calculated from the following linear equation:

*y* = 6.728*x* + 1.891 (*R*^2^ = 0.997) (4)

### 2.8. Statistical Analysis

To characterize the effect of each of the explanatory variables (temperature, pH, extract protein concentration, and substrate concentration) on the speed of absorbance (proteolysis rate), we separately carried out the following analysis for each explanatory variable: firstly, we estimated a separate linear regression of the absorbance against the reaction time at each level of the explanatory variable. From each of these models, we obtained a slope (the proteolysis rate) with its standard error. We plotted these rates (with their confidence intervals) against the different levels of the explanatory variable, and we fitted linear regression models with quadratic and cubic terms (or a Michaelis–Menten saturation curve for the substrate concentration). To study the hydrolysis time effect on caseinolytic rate, we fitted a cubic polynomial of the concentration of peptides and the proteolysis rate over time.

These models were developed using the statistical software R (version 3.3.1) using the base statistical package [28]. Parameters of V_max_ and K_m_ were calculated with the program Statistica version 10.0 (StatSoft, Hamburg, Germany).

## 3. Results

### 3.1. Temperature Effect on Proteolysis

**Figure 1:**
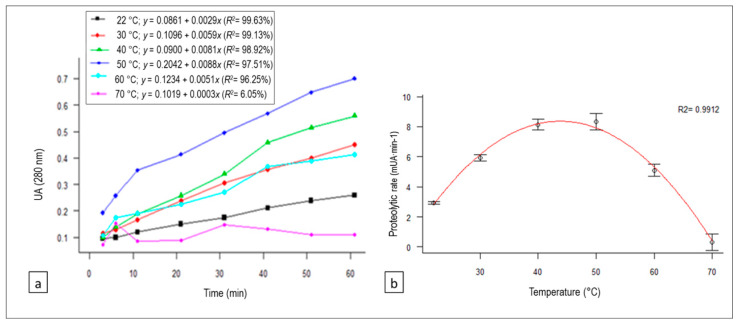
(**a**) Variation of absorbance (which correlates with proteolytic activity) with reaction time for each of the temperatures tested, as well as their regression equations and determination coefficients (*R*^2^), for the proteolytic activity linear trend of the *Cynara scolymus* L. extract on bovine casein. (**b**) Quadratic regression model of the temperature effect on the proteolysis rate of the *C. scolymus* L. extract on bovine casein.

The relationships between absorbance and reaction time at each of the temperatures studied (regression equations and *R*^2^) are shown in Figure 1a. Proteolysis rates were derived from each equation (the coefficients of *x*), and they are plotted with their confidence intervals against the different temperature levels in Figure 1b. A quadratic regression model was fitted, with the proteolysis rate (PR; expressed in mUAbs∙min^−1^) varying as a function of temperature in °C (T) according to the following equation:

PR (T) = −13.92 + 1.02T − 0.01T^2^ (*R*^2^ = 0.9912, *p*-value = 0.0008). (5)

The optimal temperature range for the proteolysis of bovine casein with the artichoke extract was 40–50 °C.

### 3.2. pH Effect on Proteolysis

**Figure 2:**
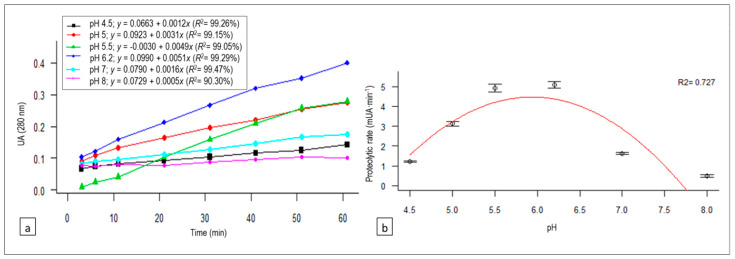
(**a**) Variation of absorbance (which correlates with proteolytic activity) with reaction time for each of the pH values tested, as well as their regression equations and determination coefficients (*R*^2^), for the proteolytic activity linear trend of the *C. scolymus* L. extract on bovine casein. (**b**) Quadratic regression model of the pH effect on the proteolysis rate of the *C. scolymus* L. extract on bovine casein.

The relationship between absorbance and reaction time (linear regression equations and *R*^2^) at each of the pH levels studied is represented in Figure 2a. The proteolysis rates (PR; expressed in mUAbs∙min^−1^) derived from each equation (the coefficients of *x*) are plotted against the different pH levels in Figure 2b, and the quadratic regression fitted was as follows:

PR (pH) = −45.43 + 16.81 pH − 1.42 pH^2^ (*R*^2^ = 0.727, *p*-value = 0.1426). (6)

The enzymatic activity of artichoke flower extract was influenced by the pH value, showing a greater proteolytic activity between pH 5.5 and 6.2.

### 3.3. *C. scolymus* L. Flower Extract Concentration Effect on Proteolysis

**Figure 3:**
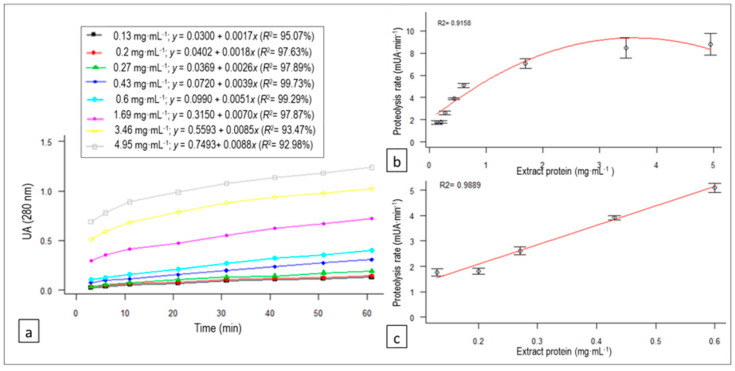
(**a**) Variation of absorbance (which correlates with proteolytic activity) with reaction time for each of the *C. scolymus* L. extract protein concentrations tested, as well as their regression equations and determination coefficients (*R*^2^), for the proteolytic activity linear trend of the *C. scolymus* L. extract on bovine casein. (**b**) Quadratic regression model of the extract protein concentration effect on the proteolysis rate of the *C. scolymus* L. extract on bovine casein. (**c**) Linear relationship between the *C. scolymus* L. extract protein concentration in the 0.13–0.6 mg·mL^−1^ range selected and proteolysis rate.

The relationship between absorbance and reaction time (linear regression equations and *R*^2^) at each of the studied concentrations of the protein extract inoculums is represented in Figure 3a. The proteolysis rates (PR; expressed in mUAbs∙min^−1^) derived from each equation (the coefficients of *x*) are plotted against different concentrations of the protein extract in Figure 3b, and the following quadratic regression model was fitted as a function of the protein concentration of the enzymatic extract in the inoculum (E):

PR (E) = 1.96 + 4.11E − 0.57E^2^ (*R*^2^ = 0.9158, *p*-value = 0.0021). (7)

In Figure 3c, we zoom in on the plot showing that, for concentrations of protein extract between 0.13 and 0.6 mg·mL^−1^, a simple linear model would generate a sufficiently good fit with *R*^2^ = 0.9889.

### 3.4. Substrate Concentration Effect: V_max_ and K_m_ Estimation

**Figure 4:**
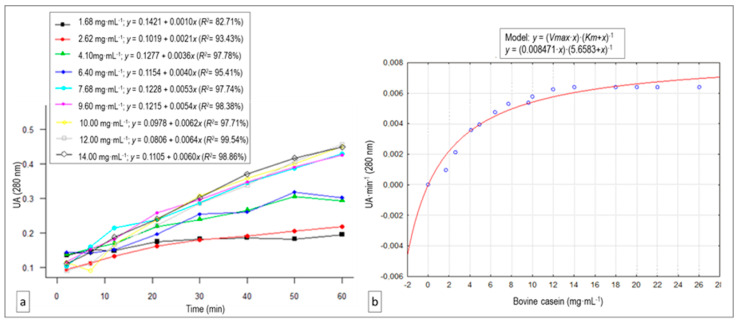
(**a**) Variation of absorbance (which correlates with proteolytic activity) with reaction time for each of the casein concentrations tested, as well as their regression equations and determination coefficients (*R*^2^), for the proteolytic activity linear trend of the *C. scolymus* L. extract on bovine casein. (**b**) Michaelis–Menten saturation curve for the enzyme reaction, showing the relationship between the bovine casein concentration and proteolysis rate (V_max_ and K_m_ estimation).

The relationships between absorbance and reaction time at each of the casein concentrations (regression equations and *R*^2^) at a constant enzymatic extract concentration (0.6 mg protein·mL^−1^), are represented in Figure 4a. In Figure 4b, we plot the proteolysis rates against different substrate concentrations, where we can observe that the reaction speed increased as the substrate concentration rose before stabilizing at approximately 10.0 mg casein·mL^−1^. Given this pattern, we fitted a V_max_ and K_m_ value estimation. The artichoke cinarases present in the extract showed a Michaelian behavior (*R*^2^ = 0.9802) (Figure 4b). 

### 3.5. Hydrolysis Time Effect on Caseinolytic Rate: Enzymatic Activity for 24 h

**Figure 5:**
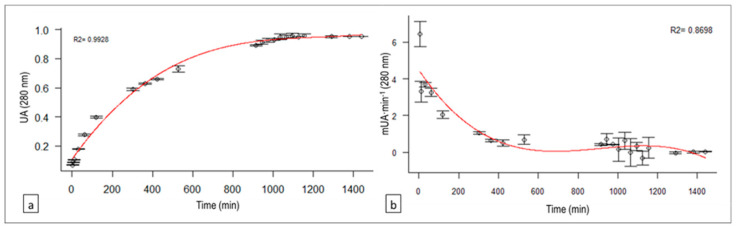
(**a**) Cubic regression model for the relationship between reaction time and absorbance (which correlates with 5% (*w*/*v*) TCA-soluble peptides) of the *C. scolymus* L. flower extract on bovine casein. (**b**) Cubic regression model for the relationship between reaction time and proteolysis velocity.

The relationship between the reaction time for 24 h and the product concentration of the enzymatic reaction (correlated with the absorbance) is represented in Figure 5a. A cubic regression model was fitted where the concentration of peptides soluble in aqueous 5% (*w*/*v*) TCA (WP) (expressed in UAbs) as a function of time in minutes (t) was as follows:

WP(t) = 0.11 + 1.94 × 10^−3^ t – 1.54 × 10^−6^ t^2^ + 4.18 × 10^−10^ t^3^ (*R^2^* = 0.9928, *p*-value < 0.0001). (8)

The water-soluble peptide concentration increased with the incubation time up to 17 h (1035 min). From this moment, the enzymatic product concentration began to stabilize.

With the reaction time, the casein proteolysis rate (PR; expressed in mUAbs∙min^−1^) decreased (absorbance difference per minute between measurements), as shown in Figure 5b. The following cubic regression model was fitted:

PR (t) = 4.48 − 1.62 × 10^−2^ t + 1.89 × 10^−5^ t^2^ − 6.95 × 10^−9^ t^3^ (*R*^2^ = 0.8698, *p*-value < 0.0001). (9)

### 3.6. ACE-I Inhibition and Antioxidant Activity of the Hydrolysates

Hydrolysis was performed for 17 h with the optimal conditions previously described (11 mg casein·mL^−1^ in distilled water (*w*/*v*) and 0.023 mg·mL^−1^ protein concentration of the artichoke flower extract, pH 6.2, 50 °C). The hydrolysates obtained showed ACE-I inhibitory activity and antioxidant capacity in vitro. Thus, 55.05% ± 6.38% ACE inhibition was observed at 0.125 mg peptide·mL^−1^. The hydrolysates exhibited a DPPH radical scavenging capacity of 30.89% ± 7.22% and a TEAC against ABTS^●+^ of 4.43 ± 0.69 mM Trolox·mg^−1^ peptide.

## 4. Discussion

The proteolytic activity of the artichoke flower extract was significantly affected by temperature (*p*-value = 0.0008), increasing the rate up to 50 °C. At 60 °C, the activity decreased, being practically nil at 70 °C. In studies performed in milk coagulation reaction characterization, it was observed that cinarase A was rapidly inactivated at 60 °C, displaying less than 20% residual activity after 60 min of incubation [14]. 

The activity of the enzymatic extract varied with the pH, showing a greater proteolysis rate between pH 5.5 and 6.2. At pH 5.0, it presented 62% of the maximum proteolytic activity observed, being lower than 35% for the rest of the pH range studied and decreasing to 10% at pH 8.0. The pH values observed to get the most efficient proteolytic activity were higher than expected, since cinarases are aspartic acid proteases, which possess an optimum pH in the acidic range close to 5.0 [29]. In studies encompassing variation of milk clotting time, Llorente et al. [13] detected an activity greater than 95% in the pH range of 4.5 to 5.5. Sidrach et al. [14] described a maximum cinarase A activity at pH 5.0, decreasing to 40% of its optimal activity at pH 3.0 and to 10% at pH 7.0. In thistle proteinases of *C. cardunculus* L., the optimal coagulation pH values observed were 5.0 [30], 5.1 [19], and 6.0 [31].

The enzymatic activity of artichoke flower extract on bovine casein varied significantly with the protein concentration of the extract (*p* for E = 0.0053; *p* for E^2^ = 0.0259), showing a greater proteolysis rate as the concentration increased. A linear regression was observed until reaching a protein concentration in the inoculum of 0.60 mg·mL^−1^ (Figure 3c). From this concentration onward, the proteolytic activity tended to stabilize. Therefore, we considered that this could be the optimal extract concentration to carry out the bovine casein hydrolysis efficiently in order to obtain bioactive peptides.

The vegetable extract showed Michaelis-Menten enzymatic kinetics. This result was similar to that obtained for wild cardoons, chymosin, or renin for milk coagulation [30,32] and in purified cinarase A [14]. The estimated V_max_ value for our crude *C. scolymus* L. flower extract on the bovine casein substrate was 8.47 ± 0.50 mUAbs∙min^−1^, and the K_m_ value was 5.658 ± 0.98 mg·mL^−1^. This last value informs us about the substrate concentration at which the enzymatic reaction takes place at the half maximum speed.

Regarding the 24-h activity curve, the major production of 5% (*w*/*v*) TCA-soluble peptides was at 17 h. A similar pattern was observed in ovine and caprine caseins hydrolyzed with A and B cardosins from *C. cardunculus* L.; however, in that case, the hydrolysis stabilization occurred between 3 and 6 h [22]. The proteolysis rate was maximum during the first minutes of incubation time. At 11 min, the speed fell to 50%. After 2 and 6 h of incubation (120 and 363 min), the speed decreased to 30% and 10% of the initial rate, respectively. At 17 h, a 99% decrease in velocity was achieved. In view of these results, prolonging the hydrolysis of casein with *C. scolymus* L. flower extract beyond 17 h of reaction time would not result in a profitable balance of time investment and benefit, since, from this moment, the proteolysis rate is practically nil.

The bovine casein hydrolysate, obtained with artichoke flower extract at the optimal conditions aforementioned, showed an ACE-I inhibitory activity of 55.05% at 0.125 mg peptide·mL^−1^, which means a higher activity than that reported by Mao et al. [33], who obtained 79.5% inhibition with a yak casein hydrolysate from alcalase while using a peptide concentration much more elevated (4 mg·mL^−1^). Similarly, a pancreatic casein hydrolysate showed 73.5% ACE inhibition at 1 mg·mL^−1^ [34], which is a concentration eight times higher than that used in our assay. The bovine casein hydrolysate from artichoke extract showed a DPPH radical scavenging activity of 30.89%. Similar results were obtained from camel casein hydrolysates with α-chymotrypsin, alcalase, and papain (37.65%, 32.25%, and 28.38%, respectively) [35]. Likewise, the bovine casein hydrolysate from *C. scolymus* extract showed antioxidant activity against ABTS^●+^ (1.09 µmol Trolox·mg^−1^ peptide). This value was higher than that exhibited by a κ-casein hydrolysate from gastrointestinal digestion in vitro (0.53 µmol·mg^−1^) [36].

It seems to be that the casein hydrolysates, obtained through the action of the *C. scolymus* L. flower extract, showed potent ACE inhibition and antioxidant activity in vitro. This may be due to the fact that the aspartic proteinases of several flower species of the *Cynara* genus have a high proteolytic action that leads to an intense casein fragmentation [37], producing small peptides. In addition, the use of these enzymes as vegetable coagulant in cheese production resulted in the formation of a greater amount of hydrophobic peptides compared to enzymes of animal or microbial origin [15,16,38]. In fact, the peptides with hydrophobic residues are those that show the highest ACE-I inhibitory activity [39]. Furthermore, peptides with hydrophobic amino acids have an important role in the free radical scavenging activity and could improve the antioxidant capacity of the hydrolysates [40,41].

## 5. Conclusions

The proteases (cinarases) present in artichoke flower extracts showed a high proteolytic activity on bovine casein. The optimal hydrolytic conditions for obtaining peptides with the *C. scolymus* L. crude extract were as follows: pH 6.2, 50 °C, 0.023 mg·mL^−1^ protein concentration of the extract, and a hydrolysis time of 17 h. As a result, a K_m_ value of 5.66 mg·mL^−1^ and a V_max_ of 8.47 mUAbs·min^−1^ were reported. 

The casein hydrolysates obtained under these conditions contained peptides with antioxidant and antihypertensive activity in vitro. Therefore, it could be possible to obtain bioactive peptides from bovine casein with extracts of *C. scolymus* flowers which remain as agricultural residue in artichoke crops.
